# Sports Activity with Ankle Osteoarthritis and Total Ankle Arthroplasty

**DOI:** 10.3390/jcm13237099

**Published:** 2024-11-24

**Authors:** Simone Santini, Andrea Marinozzi, Adrian J. Talia, Alejandro Herrera-Rodríguez, Mario Herrera-Pérez, Victor Valderrabano

**Affiliations:** 1 Swiss Ortho Center, Swiss Medical Network, Schmerzklinik Basel, 4010 Basel, Switzerland; s.santini@unicampus.it (S.S.); adrian@adriantalia.com.au (A.J.T.); 2 Department of Orthopaedic and Trauma Surgery, Fondazione Policlinico Campus Bio-Medico, Via Alvaro del Portillo, 200, 00128 Roma, Italy; a.marinozzi@policlinicocampus.it; 3 Research Unit of Orthopaedic and Trauma Surgery, Università Campus Bio-Medico di Roma, Via Alvaro del Portillo, 21, 00128 Rome, Italy; 4 Department of Orthopaedic Surgery, Western Health, Footscray Hospital, Gordon Street, Footscray, VIC 3011, Australia; 5 Foot and Ankle Unit, Orthopaedic Department, Universidad de La Laguna, 38200 San Cristóbal de La Laguna, Spainherrera42@gmail.com (M.H.-P.); 6 Faculty of Medicine, University of Basel, 4001 Basel, Switzerland

**Keywords:** ankle, foot, ankle osteoarthritis, total ankle arthroplasty, sports activity

## Abstract

**Background/Objectives:** The interest in performing total ankle arthroplasty (TAA) to address end-stage ankle osteoarthritis (OA) is continuously growing. Sports activity plays an important role in our world. The literature is sparse regarding return-to-sports activity following TAA. The levels and types of sports in TAA are rarely reported. The purpose of this prospective case series study is to investigate sports activity in ankle osteoarthritis (OA) and TAA in terms of rate, frequency, type, and clinical outcomes with a minimum 2 years of follow-up after surgery. **Methods:** A total of 103 patients (105 implants, 52 female, and 51 male), mean age 60.5 years (range, 23–84 years) with end-stage ankle OA were treated using a three-component, uncemented, mobile-bearing VANTAGE Total Ankle System. The mean follow-up was 2.9 years (range, 2–5 years). Visual Analogic Scale Pain Score (VAS, 0–10 points), Ankle Dorsiflexion/Plantarflexion (DF/PF) range of motion (ROM; degrees), functional American Orthopaedic Foot and Ankle Society (AOFAS) Ankle/Hindfoot Score (0–100 points), Subjective Patients’ Satisfaction Score (0–10 points), Sports Activity Rate, Sports Frequency Score, and sports type were assessed. **Results:** The mean preoperative VAS Pain Score was 6.7 points (range, 3–10 points) and 0.2 points for postoperative (range, 0–3 points) (*p* < 0.001). The mean DF/PF ROM was 24.9° preoperative (range, 0–60°) and 52.9° postoperative (range, 15–85°) (*p* < 0.001). The mean preoperative functional AOFAS Ankle/Hindfoot Score was 39.5 points (range, 4–57 points) and 97.8 points for postoperative (range, 75–100 points) (*p* < 0.001). The mean postoperative Subjective Patients’ Satisfaction Score was 9.7 points (range, 7–10 points). The preoperative Sports Activity Rate was 31.1%, with 85.4% for postoperative (*p* < 0.001). All the groups exhibited substantial Sports Frequency Score increases (*p* < 0.001). The most practised sports were hiking, biking, fitness, and swimming. **Conclusions:** total ankle arthroplasty (TAA) is an effective treatment for end-stage ankle OA. TAA facilitates a noteworthy increase in sports activity. This research offers important sports insights to patients with ankle OA and TAA.

## 1. Introduction

Osteoarthritis (OA) is the most prevalent type of arthritis and a major cause of pain and disability across the globe [1,2,3,4,5,6]. It is a long-term condition that encompasses various disorders leading to the functional and structural breakdown of synovial joints. This condition involves the deterioration and wear of articular cartilage, changes in subchondral bone, an inflammatory response in the synovium, and abnormal growth of bone and cartilage [7,8,9]. Key features of OA include pain, stiffness, decreased function and mobility, and a diminished quality of life, all of which contribute to functional disability.

OA is an increasing issue globally [10,11]. The Global Burden of Disease report estimates that more than 527 million people worldwide are affected by OA [11,12]. OA can be triggered by various factors, such as obesity, sports-related activities, genetic predispositions, previous injuries, occupational demands, and abnormalities in joint anatomy [13,14,15,16,17]. Additionally, osteoarthritis poses a significant socioeconomic challenge, with costs estimated to reach as high as 2.5% of the gross domestic product [18]. As the population ages, the prevalence of OA is expected to rise further.

About 1% of the world’s population are affected by painful end-stage ankle OA [19]. It has been shown to be a limiting condition in both vocation and recreation and affects patients who experience a level of pain and debilitation at least as severe as the ones who suffer from hip OA, congestive heart failure, and end-stage kidney disease [20,21,22].

Trauma is the aetiology of ankle OA in at least 80% of cases, while primary OA and other secondary aetiology represent less than 20% [23,24,25]. Whilst ankle fractures are the primary culprit, a significant proportion of patients have chronic instability as the causative factor in their degenerate ankle joint [26,27]. As a consequence, ankle OA patients are relatively young, and their demand for a return to sport is high [28,29]. Ankle arthrodesis remains the most common operation for end-stage ankle OA; however, with improved techniques and implants, total ankle arthroplasty (TAA) is becoming more common each year [30,31,32,33].

The most recent studies including large multi-centre randomized controlled trials show that TAA is not inferior to ankle arthrodesis in terms of functional outcomes [34,35]. It also offers a better health-related quality of life and gait pattern with equal complication and revision rates [36]. The American Orthopaedic Foot and Ankle Society (AOFAS) endorses TAA as a viable choice for addressing ankle OA (AOFAS Statement 2022).

However, there is still a lack of evidence regarding TAA and sports. Outcomes, levels, and types of sports have not previously been investigated [37,38,39].

Therefore, this prospective study aimed to investigate sports activity in ankle OA and following TAA in terms of activity amount, sports type, and the clinical and radiographic outcomes at a minimum follow-up of 2 years after TAA surgery.

## 2. Methods

In this prospective case series study, 103 consecutive patients (52 female and 51 male; 105 implants; 2 bilateral cases) were enrolled at the Centre of the senior author. The mean patient age was 60.5 years (range, 23–84 years). All patients received a cementless, three-component mobile-bearing VANTAGE Total Ankle Arthroplasty (Exactech, Gainesville, FL, USA) for end-stage ankle OA with any aetiology. Demographic data are shown in Table 1.

**Figure 1:**
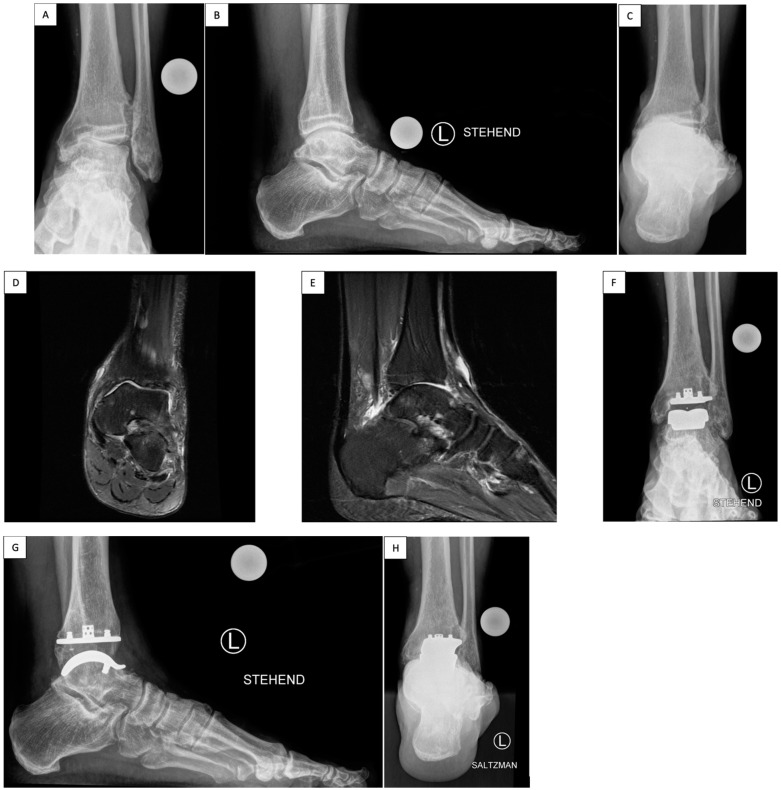
Sports-active patient with VANTAGE Total Ankle Arthroplasty mobile-bearing system. A 59-year-old patient suffered from post-traumatic ligamentous end-stage varus ankle osteoarthritis (OA) with chronic lateral ligament ankle instability and peroneus brevis tendon lesion (**A**–**C**, pre-op). The preoperative MR images show the medial predominantly ankle OA (**D**,**E**, pre-op). The surgery comprised TAA implantation, deltoid release, lateral ankle ligament reconstruction, and peroneus longus (PL)-to-brevis (PB) tendon transfer. After TAA surgery at recent 3-year follow-up he reached an AOFAS Ankle/Hindfoot Score of 100, VAS Pain Score of 0, Dorsiflexion/Plantarflexion 25-0-40, and Inversion/Eversion 30-0-10 (**F**–**H**); could return to a sports level of >5 h/week including hiking over 10 km and skiing; and obtained a Subjective Patients’ Satisfaction Score of 10 points (very satisfied). He describes a sense of the “forgotten joint” level. The radiological examination shows at 3-year follow-up a well-osteointegrated TAA with no signs of lucency or loosening.

As part of the study, patients were analysed both pre- and postoperatively (Figure 1). Inclusion criteria were painful end-stage ankle OA and meeting general TAA criteria such as having good bone quality, normal vascular condition, ligamentous stability, and good neuromuscular control. Exclusion criteria were neuroarthropathy (e.g., Charcot ankle), active or recent infection, poorly controlled or complicated diabetes (e.g., peripheral neuropathy or renal disease), large areas of avascular necrosis of the talus, severe benign joint hypermobility syndrome, severe malalignment, substantial soft tissue problems around the ankle, and sensory or motor dysfunction in the foot or leg.

Study questionnaires were administered preoperatively, and then at 6 weeks, 6 months, 1 year, 2 years, 3 years, and 5 years postoperatively. Patients enrolled in the current cohort had a minimum of 2 years of follow-up post-surgery. The mean follow-up period in this study was 2.9 years, ranging from 2 to 5 years. Pain levels were assessed using a visual analogue scale (VAS), where 0 indicated no pain and 10 indicated the most extreme pain imaginable. Patients’ satisfaction was evaluated by the Subjective Patients’ Satisfaction Score on a scale ranging from excellent (10 points) to none (0 points). The functional range of motion (ROM) of the ankle joint in the sagittal plane was measured using a goniometer in degrees for dorsiflexion (DF) and plantarflexion (PF) with the patient standing. The American Orthopaedic Foot and Ankle Society (AOFAS) Ankle/Hindfoot Score, subdomains of which consider pain, function, and alignment, was used to assess the overall clinical–functional status, scoring between 0 and 100 points [40].

Each patient’s sports activity level was documented before the surgery and at the TAA follow-up using the Sports Activity Rate (number of sports-active patients), and the Sports Frequency Score (a scale from level 0 (no sports activity) to level 4 (Elite sports activity)) [37]. Further, all reported sports types were recorded.

Standard weight-bearing radiographs were taken preoperatively and during follow-up in four projections (anteroposterior (AP) ankle, lateral, and AP foot, and hindfoot alignment view), as demonstrated in Figure 1. Radiolucency was evaluated and defined as a gap narrower than 2 mm between the TAA implant and the bone, which was not visible on the initial radiographs taken within the first 6 weeks after the surgery [41]. Osteolysis was defined as a clearly marked, non-linear lytic lesion with a width of 2 mm or more [41]. The potential presence of periarticular ossifications (PAOs) was assessed and graded according to a previously published study on heterotopic ossification after TAA on a scale of 0 to 4 [42].

Ethics approval was sought and granted from the Institutional Review Board (IRB), all participants gave informed consent, and the study followed the guidelines outlined in the World Medical Association Declaration of Helsinki. Data were analysed using a paired Student *t*-test, with a significance level set at *p* < 0.05 and the Chi-squared test.

### 2.1. Surgical Technique

The VANTAGE Total Ankle Arthroplasty mobile mobile-bearing system is implanted according to the technique previously described by Valderrabano et al. [43]. The patient is positioned supine with a bump under the ipsilateral buttock to prevent external rotation of the limb, with a thigh tourniquet set at 250 mmHg. An anterior midline skin incision is performed, caution is essential beneath the skin to prevent any harm to the superficial peroneal nerve or its branches. The extensor reticulum is incised, and the interval between the tibialis anterior (TA) and the extensor hallucis longus (EHL) is developed, taking care not to injure the neurovascular bundle that lies under the EHL, the neurovascular bundle is taken laterally and protected throughout the procedure. The ventral ankle capsule is opened, and the anterior osteophytes are removed with a chisel.

A pin is inserted perpendicular to the tibial axis percutaneously into the tibial tubercule. The tibial alignment guide is then positioned and secured in the appropriate alignment in terms of rotation, varus/valgus, slope, and resection height; this is verified through fluoroscopy. The tibial cut is then made though the secured tibial cutting guide. To safeguard the medial and lateral malleoli during the tibial cut, it is possible to insert a pin at either end of the tibial cutting guide.

The cut tibial bone is then removed and the talar cutting guide is positioned. The block is extended distally as much as possible to create physiological tension in the ligaments and then fixed with 2 pins. The talar dome cut is performed and the cut bone is removed. The appropriate-sized talar cutting block is then positioned, and the anterior and posterior talar surfaces are prepared. The cutting block and the bone pieces are removed. A curved rasp is employed to smooth the talar bone into a rounded, curved shape, resulting in a dome-like surface. The talar trial position is checked under fluoroscopy, and the talar peg holes are drilled.

The AP sizing tool measures the tibia, guiding the choice of the tibial punch guide size. A lateral fluoroscopic view confirms posterior tibia coverage and the cage and the three peg holes are prepared.

The definitive components are then implanted. Trial inlays help to assess the correct polyethylene thickness to provide a good range of motion and physiological ligament strain. Now, the definitive polyethylene component is implanted. A drain is positioned in the joint. The layers are sutured closed. A sterile dressing is applied (Figure 1).

### 2.2. Postoperative Care

After surgery, in the first days the ankle/leg is elevated for the majority of the day. Physiotherapy promotes lymphatic drainage, and Achilles tendon stretching. The patient is encouraged to perform “skiing exercises” at the bedside to stretch the gastrocnemius-soleus muscles, which has an added benefit of improving TAA press fit. Patients were asked to perform these ankle dorsiflexion exercises three to four times a day for 5 min. The patients use a walker and crutches to walk long distances or outside. Short distances at home (e.g., in the bathroom or room), the patients are allowed to walk barefoot under full-weight bearing. Complete weight-bearing is permitted right away for isolated ankle arthroplasty. Partial weight-bearing 15 kg for 6 weeks is instructed for cases where additional procedures are performed, such as additional hindfoot osteotomies or ligament reconstruction.

### 2.3. Results

The aetiology of ankle OA was as follows: 75 cases (77.4%) had post-traumatic OA, 14 cases (13.3%) had secondary OA, and 16 cases (15.2%) had primary OA.

At a mean follow-up of 2.9 years, the VAS Pain Score improved from a mean of 6.7 (range 3–10) preoperatively to 0.2 (range 0–3) postoperatively (*p* < 0.001). The Subjective Patients’ Satisfaction Score with TAA indicated at follow-up a mean value of 9.7 points (range, 7–10). The mean DF/PF ROM increased from 24.9° (range 0–60°) preoperatively to 52.9° (range 15–85°) postoperatively, *p* < 0.001). The mean preoperative AOFAS Ankle/Hindfoot Score was 39.5 points (range, 4–57 points), which improved to 97.8 points (range, 75–100 points) at follow-up (*p* < 0.001). Table 2 displays the results stratified by group and in total.

**Figure 2:**
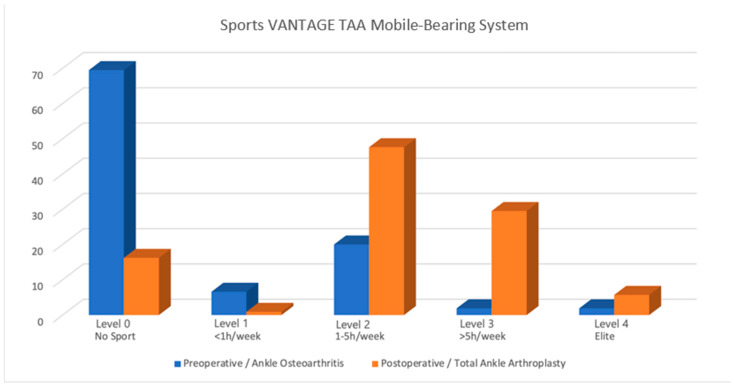
Sports Frequency Score in ankle osteoarthritis and total ankle arthroplasty. *p* < 0.001.

Prior to TAA, 32 patients (31.1%) were sports-active, which increased to 88 patients post-TAA (85.4%), representing an increase of 54.3% (*p* < 0.001). All the groups exhibited significant Sports Activity Rate increases (*p* < 0.001). The distribution of the Sports Frequency Score differed significantly for TAA compared to the preoperative arthritic condition (Table 3; *p* < 0.001). After TAA, there was a noteworthy increase in cases classified as levels 1 to 3 of the Sports Frequency Score (*p* < 0.001), with 6 of the patients achieving their grade 4 (elite activity level) again (an increase from only 2 patients before). Sporting outcomes are shown in Table 3, stratified by group and in total; a graphical representation of the weekly sports engagement post TAA is shown in Figure 2.

All patients actively involved in sports before surgery maintained or increased their sports activity post-TAA except one. Among patients not engaged in sports before surgery, 73.4% (n = 52) were engaged in sporting activity after TAA surgery (*p* < 0.001), and the patients fell mainly into levels 1 to 3 on the sports frequency score. The most common sporting activity preoperatively were cycling (29.6%), swimming (17.7%), fitness (15.5%), and walking (11.4%). Postoperatively, the most common sports were hiking (43%), cycling (15%), fitness (11.7%), and swimming (8%) (Table 4).

In this study, there were no revisions of the tibiotalar components, no explantations, and no infections occurred. In one case (0.9%), residual medial ankle instability after 2 years necessitated medial deltoid ligament reconstruction, thicker polyethylene inlay, and medial periarticular ossification (PAO) resection. In one case (0.9%) of a professional mountain guide (post-traumatic ankle OA, smoker), a stress fracture of the medial malleolus was addressed by bone grafting and plate open reduction, internal fixation (ORIF).

Radiological changes were identified in seven cases (6.6%); none of these patients were symptomatic. Non-progressive radiolucency was identified in three cases (2.8%) around the tibia component, with no radiolucencies observed adjacent to the talar component. All three cases were post-traumatic ankle OA cases. Further, four cases had isolated tibial cysts (3.8%) at the tibial bone implant interface: two cases with a tibia cyst of 5mm antero-lateral (the first in a professional mountain guide, post-traumatic ankle OA, smoker, medial malleolus stress fracture ORIF; the latter in a post-traumatic ankle OA); a case with a tibia cyst of 5mm anterocentral (post-traumatic ankle OA); a case with tibia cyst 7 mm anterocentral (secondary ankle OA after triple arthrodesis). No cysts were seen at the talar bone–implant interface.

Furthermore, we radiologically identified PAO in a total of 16 cases (15.2%). The mean PAO score was 1.6 (range 1–2).

## 3. Discussion

This research demonstrates that patients who underwent total ankle arthroplasty (TAA) demonstrated improved outcomes in terms of the VAS Pain Score, overall subjective satisfaction, sagittal ROM, AOFAS Ankle/Hindfoot Score, and their engagement in sports activities at an average follow-up duration of 2.9 years. Significant increases in the Sports Activity Rate (number of sports-active patients) and Sports Frequency Score have been found. This is the first paper in the literature reporting sports activity with the three-component mobile-bearing VANTAGE TAA system.

Unsurprisingly, 68.3% of the patients did not participate in any sports activities before their surgery. Of these ankle osteoarthritis patients, the remaining 31.7% participated in sports activities, primarily falling into moderate sports at level grade 2 (1–5 h/week), as indicated in Table 3. These patients typically opted for low-impact sports like biking and swimming, as listed in Table 4.

The VANTAGE TAA Mobile-Bearing System shows promising results in terms of pain improvement (VAS), function (AOFAS), ROM, and revision rate compared to other prostheses in the ankle arthroplasty literature. In 2021, Cho et al. conducted a prospective comparative study reporting the 3-year clinical outcomes of the mobile-bearing prosthesis Zenith (Corin, Cirencester, UK) implanted in 64 patients affected by end-stage ankle OA (45 post-traumatic cases and 19 rheumatoid cases). They identified no significant differences in outcome based on the aetiology, which is consistent with our cohort. A general improvement in both groups’ average AOFAS scores has been recorded (39.4 ± 10.2 was the mean preoperative value, and 82.8 ± 14 was the mean postoperative one). The average Foot and Ankle Outcome Score (FAOS) increased from a preoperative value of 35.1 ± 11.2 to 72.7 ± 16.2 postoperatively. The ROM (dorsi-plantarflexion) improved from a preop mean of 38.5° to a mean of 41.2° postoperatively. Their radiological assessment identified late postoperative complications in 28 patients. These complications included implant loosening, osteolysis, polyethylene (PE) insert subluxation, heterotopic ossification, impingement from soft tissue or bone spurs, and OA development in the nearby joints. Four patients (8.9%) from the OA group and two patients (10.5%) from the RA group required revision surgery, involving the replacement of one or more components. Among the six revision surgeries performed, three involved the revision of the talar component and bone graft, while the other three cases involved the exchange of the PE insert and bone graft [44].

Van Haecke and colleagues examined 94 Hintegra prostheses with an average follow-up period of 81 months (range, 19–124 months). The survival rate without the need for revision surgery was 76%, and the rate without explantation was 92%, with 10 cases requiring curettage and 5 undergoing explantation. The average AOFAS score improved from 41.8 (range, 21–69) to 77.5 (range, 24–100). Overall, 75% of the patients experienced either no pain or only mild pain. The average ROM value was 23.5° (range, 5–48°). A total of 54.6% of cases exhibited posterior tibial calcification during the follow-up period. The risk of developing severe cysts (>1 cm) on computed tomography (CT) scans was 36% at a mean follow-up duration of 77 months (range, 18–123) [45].

The relationship between sport and TAA has been previously investigated in the literature. Recently, Usuelli et al. found a significant increase in sports activity at a 12-month follow-up after the implantation of Hintegra total ankle prosthesis in 76 patients: prior to the surgical procedure, 11.7% of the patients (n = 9) participated in sports, and following the surgery, this percentage increased to 49.4% (n = 38). Jogging, dancing, biking, and skiing were the patients’ most pursued sports activities. However, a small number of them (14 patients in total) engaged in high-impact sports, including jogging (13 patients) and martial arts (1 patient), even though they had medical recommendation to avoid these activities [39].

In 2009, Naal and colleagues conducted a study examining the sports and recreational activity participation of 101 patients before and after receiving a three-component uncemented “mobility” prosthesis, with an average follow-up period of 3.7 years. Prior to the surgery, 62.4% of the patients were engaged in sports, and after the operation, 66.3% remained active (*p* = 0.56), indicating no significant change. Post-TAA activities included swimming, cycling, and engaging in fitness or weight training. A total of 65% of the cohort reported that the surgery had enhanced their sports capabilities [46].

Valderrabano et al. found a significant increase in sports engagement in a consecutive series of 147 cases (152 ankles) who received a Hintegra implant: prior to the surgical procedure, 36% of the patients participated in sports, and following the surgery, this proportion increased to 56%. The top three most common sports activities included hiking, cycling, and swimming [37].

In this investigation, the percentage of individuals engaging in sports following TAA was 87.1%, and hiking was the most frequently reported sports activity post-TAA. Based on the published literature, low-impact activities have been recommended to our cohort of patients [37]. Nevertheless, several patients in our cohort were practising running, basketball, skiing, paragliding, dancing, or even multiple sports, and no relationship has been observed between radiological changes, revision surgery, and engagement in sports activities (Table 4). We share the viewpoint that impact sports could positively impact the connection between bone and implant, possibly promoting bone ingrowth as long as the applied forces remain below a certain threshold, which is yet to be determined. Moreover, sports activities might contribute to mitigating muscle loss, which is common in ankle arthritis and could enhance the strength of the leg muscles [47]. Consequently, this could decrease the occurrence of excessive joint reaction forces during movement [37].

The improved success achieved with joint arthroplasty, particularly in the case of knee and hip joints and concerns regarding the long-term outcomes following ankle arthrodesis, have led to a renewed interest in TAA [48,49,50]. Current-generation TAA systems have achieved the goal of replicating normal ankle function, maintaining normal joint biomechanics, ensuring physiological ligament stability and proper mechanical alignment [51]. Similarly to total hip replacement (THR) and total knee replacement (TKR), the positive outcomes and increased long-term viability have encouraged patients and surgeons to consider TAA as an effective treatment for end-stage ankle OA, ultimately helping them regain better functionality and even engage in sports [37].

Engagement in sports ranges from 29% to 56% following THR and between 34% and 77% after TKR [52,53]. In a 2023 systematic review, the average rate of sports activity after TAA was found to be 61.9% (ranging from 49.4% to 76%), which is consistent with the rates seen in other joint arthroplasty procedures [54].

The rate at which our patient cohort participated in sports was higher. This may be due to the chosen implant. An ankle prosthesis meant for sport needs specific features: high rotational and shear force resistance, stability through gait, the ability to convert shear forces to compressive forces, and high polyethylene resistance to stress forces and to dislocation at extreme range of motion. The mobile-bearing VANTAGE TAA system is the current fifth generation of ankle prosthesis, which has been developed with a particular focus on activity. The design of the tibial component is intended to match the natural cortical anatomy of the distal tibia; it is anatomically contoured to prevent interference with the fibula and minimize the risk of tibial component impingement, a common source of post-TAA pain [55]. The design also prevents any compromise to the anterior tibial cortex, safeguarding bone integrity and avoiding the entry of wear particles into the bone–implant interface. The fixation of the tibial component involves a titanium-plasma-coated surface, a centrally placed press-fit cage, and three vertical pegs. This configuration ensures stability and axial compression, and prevents rotation at the bone–implant interface, thereby mitigating the risk of loosening. The talar component is anatomically shaped, featuring a conical form and a bicondylar articulating surface. This was designed to mimic the native biomechanics of the ankle joint during walking. The bone–implant interface employs an arc-shaped titanium-plasma-coated contact area, preserving bone and acknowledging the altered talar shape [56]. This curved design allows compressive forces to remain perpendicular to the bone surface throughout the ROM, offering stability and thereby reducing stress-shielding [57]. The talar component is further secured with two pegs for rotational and mediolateral stability, while a small anterior flange prevents potential loosening or migration. The moulded polyethylene insert’s bicondylar articular surface guarantees the absence of machined fibrils at the interface and maintains fracture toughness. The fit between the polyethylene liner and talus helps distribute stress across the surface, reducing peak forces and minimizing the risk of polyethylene wear [58].

The question of whether mobile-bearing total ankle arthroplasty (MB-TAA) or fixed-bearing total ankle arthroplasty (FB-TAA) is better remains a subject of ongoing discussion. Long-term success has been observed in the case of MB-TAA, while the newer generation FB-TAA has demonstrated favourable outcomes during medium-term follow-up, but long-term (>10 year) results are so far not available [34,59].

Nunley et al. compared patient satisfaction, functional outcomes, and X-ray findings when using the mobile-bearing STAR and the fixed-bearing Salto-Talaris implants. Data gathering encompassed the VAS Pain Score, Short Form 36 questionnaire, Foot and Ankle Disability Index, Short Musculoskeletal Functional Assessment, and AOFAS Hindfoot score. Even though no specific sports analysis was performed, their findings indicated that patient-reported and clinical outcomes were positive for both implant designs, and there was no notable disparity in clinical improvement between the two types of implants. The occurrence of lucency/cyst formation in the tibial component was comparable. However, the MB-TAA system exhibited more extensive lucency/cyst formation in the talus and experienced greater subsidence in both the tibial and talar components [60].

In a direct comparison to the prosthesis used in our study, Henry JK et al. recently investigated the outcome of the fixed-bearing Vantage. After a follow-up period averaging 2.81 years, implant failure was registered in 10 cases (5.8%). Among these, six exhibited loosening of the tibial component. Of the other four, one was attributed to periprosthetic joint infection, one to loosening of the talar component, and two to the loosening of both the tibial and talar components. Asymptomatic peri-implant lucency/subsidence was observed in 20.1% of ankles, with the majority involving the tibial component, specifically 25 cases. Comparing these results to our data, MB-Vantage shows superior outcomes regarding complications and implant failures, compared to its fixed bearing counterpart [59].

This study is limited regarding the medium-term period of follow-up. To arrive at meaningful conclusions regarding the impact of the level of sport engagement and different types of sports on TAA, it is essential to assess this cohort over a longer period. The study focuses on patients receiving the VANTAGE Total Ankle System, which may limit the applicability of the results to other implant systems or surgical techniques. A broader comparison of different implants could provide more comprehensive insights. However, by focusing on this well-defined patient group, we were able to perform a detailed and controlled evaluation of sports activity post-TAA, which might not have been possible with a more heterogeneous cohort or using multiple implant systems. Additionally, the sports activity score only reflects the frequency of sports participation and does not account for the varying physical demands associated with different activities.

## 4. Conclusions

Total ankle arthroplasty (TAA) is an effective treatment for end-stage ankle OA. TAA significantly improves the overall rate of sports participation, enhancing the quality of life for these patients. This research offers important sports insights to patients with ankle OA and TAA. These findings are also valuable to orthopaedic surgeons when advising ankle OA and TAA patients regarding sports activities. Patients undergoing TAA treatment who are engaging in high-impact activities show no correlation with revision rate or radiological changes at the recent follow-up time. Nonetheless, more studies are still needed to provide surgeons with more robust evidence upon which to base their recommendations for guiding their TAA patients in performing safe sports activities.

## Figures and Tables

**Table 1:** Demographic data.

Total Patients	n = 103
Total TAA Cases	n = 105
Women	n = 52 (50.5%)
Men	n = 51 (49.5%)
Age, mean (range)	60.5 years (23–84 years)
Follow-up, mean (range)	2.9 years (2–5 years)

TAA: total ankle arthroplasty.

**Table 2:** Clinical outcomes.

	VAS Pain Score				AOFAS Score			
	Preoperative/Ankle Osteoarthritis		Postoperative/Total Ankle Arthroplasty		Preoperative/Ankle Osteoarthritis		Postoperative/Total Ankle Arthroplasty	
Etiology	Mean	Range	Mean	Range	Mean	Range	Mean	Range
Posttraumatic Osteoarthritis (n = 75, 77.4%)	6.6	3–10	0.2 *p* < 0.001	0–3	39.5	4–72	97.8 *p* < 0.001	75-100
Primary Osteoarthritis (n = 16, 15.2%)	6.7	5–10	0 *p* < 0.001	0	39.5	5–55	97.6 *p* < 0.001	89-100
Secondary Osteoarthritis (n = 14, 13.3%)	6.7	5–8	0.2 *p* < 0.001	0–3	39.4	12–57	97.6 *p* < 0.001	87-100
Total (n = 105)	6.7	3–10	0.2 *p <* 0.0001	0-3	39.5	4–57	97.8 *p* < 0.001	75–100

AOFAS: American Orthopaedic Foot and Ankle Society; AOFAS Ankle/Hindfoot Score; VAS: Visual Analogue Scale. *p* value indicates significance level from Student’s *t*-test.

**Table 3:** Sports Activity Rate and Sports Frequency Score in ankle osteoarthritis and total ankle arthroplasty.

	Preoperative/Ankle Osteoarthritis				Postoperative/Total Ankle Arthroplasty			
Sports Activity	Post-traumatic n (%)	Secondary n (%)	Primary n (%)	Total (%)	Post-traumatic(%)	Secondary (%)	Primary (%)	Total (%)
Sports Activity Rate	28 (37.3%)	3 (21.5%)	1 (6.3%)	32 (31.1)	68 (90.7%) *p* < 0.001	9 (64.3%) *p* < 0.001	11 (68.8%) *p* < 0.001	88 (85.4%) *p <* 0.001
Sports Frequency Score	Posttraumatic n (%)	Secondary n (%)	Primary n (%)	Total (%)	Posttraumatic (%)	Secondary (%)	Primary (%)	Total (%)
0/None	44	12	15	71 (68.9)	9	5	5	19 (18.5)
1/Moderate <1 h/week	6	1	0	7 (6.8)	1	0	0	1 (1)
2/Normal 1–5 h/week	20	1	0	21 (20.4)	39	6	5	50 (48.5)
3/High >5 h/week	1	0	1	2 (2)	23	3	5	31 (31)
4/Elite	1	1	0	2 (2)	5	0	1	6 (5.9)
Standard Deviation (SD)				28.34				19.30

Sports Activity Rate: number of sports-active patients; Sports Frequency Score: level 0 (none) to level 4 (elite) [37]. Post-traumatic: post-traumatic ankle osteoarthritis; Secondary: secondary ankle osteoarthritis; Primary: primary ankle osteoarthritis. *p* value indicates significance level from Chi-squared test.

**Table 4:** Sports type in ankle osteoarthritis and total ankle arthroplasty. Sports type data aligned in decreased % listing sports activity. *p* < 0.05.

List of Reported Sports Activities		
	Postoperative/ Total Ankle Arthroplasty	Preoperative/ Ankle Osteoarthritis
Types of Sport Activities	Patients (%)	Patients (%)
Hiking	66 (43)	1 (2.2)
Cycling	23 (15)	13 (29.5)
Fitness	18 (11.7)	7 (15.5)
Swimming	8 (5.2)	8 (17.7)
Walking	8 (5.2)	5 (11.4)
Skiing	6 (3.9)	1 (2.2)
Running	5 (3.3)	2 (4.4)
Dancing	3 (2)	1 (2.2)
Horseback Riding	3 (2)	2 (4.4)
Golf	2 (1.3)	1 (2.2)
Alpinism	2 (1.3)	0
Basketball	2 (1.3)	0
Hunting	2 (1.3)	1 (2.2)
Tennis	2 (1.3)	0
Powerlifting	1 (0.7)	1 (2.2)
Jump Rope	1 (0.7)	1 (2.2)
Paragliding	1 (0.7)	0
Martial Art	1 (0.7)	0
Climbing	0	1 (2.2)

## Data Availability

The data presented in this study are available on request from the corresponding author. The data are not publicly available due to ethical and privacy reasons.
